# Survivability and proliferation of microorganisms in bentonite with implication to radioactive waste geological disposal: strong effect of temperature and negligible effect of pressure

**DOI:** 10.1007/s11274-023-03849-0

**Published:** 2023-12-10

**Authors:** Deepa Bartak, Eva Bedrníková, Vlastislav Kašpar, Jakub Říha, Veronika Hlaváčková, Petr Večerník, Šárka Šachlová, Kateřina Černá

**Affiliations:** 1 Institute for Nanomaterials, Advanced Technologies and Innovation, Technical University of Liberec, Bendlova 7, 460 01 Liberec, Czech Republic; 2 Disposal processes and safety, ÚJV Řež, a. s., Hlavní 130, 250 68 Husinec, Czech Republic

**Keywords:** Bentonite suspension, Deep geological repositories, Limiting factors, Microbial activity, Microorganism survivability, Radioactive waste disposal

## Abstract

As bentonite hosts a diverse spectrum of indigenous microorganisms with the potential to influence the long-term stability of deep geological repositories, it is essential to understand the factors influencing microbial activity under repository conditions. Here, we focus on two factors, i.e., temperature and swelling pressure, using a suspension of Cerny Vrch bentonite to boost microbial activity and evaluate microbial response. Suspensions were exposed either to different pressures (10, 12 and 15 MPa; to simulate the effect of swelling pressure) or elevated temperatures (60, 70, 80 and 90 °C; to simulate the effect of cannister heating) for four weeks. Each treatment was followed by a period of anaerobic incubation at atmospheric pressure/laboratory temperature to assess microbial recovery after treatment. Microbial load and community structure were then estimated using molecular-genetic methods, with presence of living cells confirmed through microscopic analysis. Our study demonstrated that discrete application of pressure did not influence on overall microbial activity or proliferation, implying that pressure evolution during bentonite swelling is not the critical factor responsible for microbial suppression in saturated bentonites. However, pressure treatment caused significant shifts in microbial community structure. We also demonstrated that microbial activity decreased with increasing temperature, and that heat treatment strongly influenced bentonite microbial community structure, with several thermophilic taxa identified. A temperature of 90 °C proved to be limiting for microbial activity and proliferation in all bentonite suspensions. Our study emphasizes the crucial role of a deep understanding of microbial activity under repository-relevant conditions in identifying possible strategies to mitigate the microbial potential within the deep geological repository and increase its long-term stability and safety.

**Supplementary Information:**

The online version contains supplementary material available at 10.1007/s11274-023-03849-0.

## Introduction

Deep geological repositories (DGRs) are considered the most reliable solution for radioactive waste disposal globally, ensuring long-term safety by isolating hazardous radionuclides from the biosphere. This is achieved through an engineered barrier system (EBS) comprising several complementary safety functions, wherein high-level radioactive waste is buried several hundred metres underground in a stable geological environment encapsulated in metal corrosion-resistant canisters (Feiveson et al. 2011). In many EBS concepts, including that in the Czech Republic, highly compacted bentonite clay plays a crucial role, both in containing spent fuel canisters from the environment and maintaining their integrity over time and as backfill material in the disposal tunnel (Dohnálková et al. 2022). This is accomplished by preventing the passage of corrosive groundwater components and oxidants into the canister, by providing mechanical protection in the event of any rock movement, by retarding radionuclide migration and by preventing mobilisation of corrosion products in the event of canister failure (Posiva Oy 2017a). Bentonite has several fundamental characteristics that make it ideal for such a role, including its ability to self-seal, low hydraulic conductivity, high sorption capacity, swelling capacity and thermal conductivity, and its long-term stability (SKB 2010; Posiva Oy 2017a). Bentonite is predominantly composed of montmorillonite, which is responsible for its high swelling capacity following water uptake. In confined spaces, this swelling can lead to a pressure build-up (known as swelling pressure) and a reduction in initially larger pores when the compacted bentonite is saturated (SKB 2010).

As a natural material, bentonite is not sterile and hosts diverse indigenous, often spore-forming, microorganisms (Svensson et al. 2011; Engel et al. 2019), the uncontrolled metabolic activity of which can cause unwanted effects within the repository. Most importantly, microbially-influenced corrosion (MIC) of metal canisters by sulphate-reducing bacteria (Masurat et al. 2010; Bengtsson and Pedersen 2017), nitrate-reducing bacteria (Povedano-Priego et al. 2019) or acetogens (Svensson et al. 2011) can increase the risk of canister failure (Shrestha et al. 2021). Furthermore, iron-reducing bacteria can reduce structural iron in smectite, causing mineralogical changes within the bentonite, e.g. illitisation (Kim et al. 2004). Bentonite microorganisms can also play an important role by altering redox conditions through the production of gases such as carbon dioxide (CO_2_) or methane (CH_4_) as a by-product of metabolic activity, by changing geochemical conditions within the DGR or by causing an undesired pressure build-up (Stroes-Gascoyne et al. 2011). Moreover, in cases of canister failure, mobile microbes may sorb released radionuclides and act as colloids, potentially increasing the migration of radionuclides (Merroun et al. 2005).

Both the activity and proliferation of microbes can be strongly limited through bentonite compaction. Several dry/wet density thresholds for microbial activity have been determined for different bentonites, ranging from 1690 kg/m^3^ for Czech Rokle clay to over 2000 kg/m^3^ for Belgian Boom Clay (Bengtsson and Pedersen 2016, 2017; Pedersen 2017). As a consensus, various repository concepts require a dry clay density greater than 1600 kg/m^3^, and a resulting swelling pressure of at least 5 MPa (Sirpa et al. 2022). Several studies have highlighted factors that may be responsible for limiting microbial activity in saturated compacted bentonites, with the evolution of swelling pressure, restricted pore space, a decrease in water activity and limitation of nutrient diffusion all playing important roles (Bengtsson and Pedersen 2016; Pedersen 2017), though the importance of individual factors remains unresolved.

While compaction of bentonite can restrict microbial activity, both indigenous bacteria (Pedersen et al. 2000; Aoki et al. 2010) and/or cultivable bacteria introduced prior to compaction for experimental reasons (Pedersen et al. 2000; Bengtsson and Pedersen 2017), have been shown to survive as spores, even when the clay is highly compacted. The spore-forming ability of these microorganisms enables extreme tolerance to desiccation, high temperatures and salinity (Masurat et al. 2010), allowing them to survive unfavourable conditions by remaining dormant (Nicholson et al. 2000). Thus, while compaction can have a negative impact on the cultivability of indigenous microorganisms (Pedersen et al. 2000; Smart et al. 2017), it does not eradicate them, and they can rapidly germinate and regain metabolic activity when conditions become favourable. Therefore, it is essential to understand other limiting factors besides dry density that determine microbial survivability under repository conditions. One of the most important of these alternative limiting factors is temperature. Within a few years of emplacement, the surface temperature of waste canisters is expected to reach ≈ 100 °C, with temperatures within the bentonite buffer itself not expected to exceed 100 °C under most DGR concepts (Posiva Oy 2013; Posiva Oy and SKB 2017). However, studies have demonstrated that temperatures ranging from 50 to 85 °C can negatively influence microbial survivability (Lydmark and Pedersen 2011); Pedersen et al. 2000); consequently, the impact of temperature on microbial activity and proliferation must be carefully evaluated to enable reliable predictions of how microbial activity will evolve under DGR conditions.

In our study, we investigate the discrete effects of two key parameters, swelling pressure and temperature, on microbial activity in Czech Bentonite Černý Vrch (BCV) suspensions in a 6–7 week long laboratory experiment. Though bentonite suspensions do not represent systems fully comparable with compacted bentonite under DGR conditions, they can help simulate some of the microbial effects expected to occur over reasonably long time scales in future DGRs, unlike experiments in compacted bentonite that require much longer exposure times. Furthermore, bentonite suspensions may occur in DGRs where there is a massive decrease in compacted bentonite density and bentonite erosion occurs (Baik et al. 2007). In such cases, bentonite suspensions could mimic the extreme environment of such a worst-case scenario regarding bentonite buffer state. As such, bentonite suspensions represent a useful experimental system for predicting trends in bentonite buffer microbial community development (Grigoryan et al. 2018; Matschiavelli et al. 2019; Miettinen et al. 2022). Bentonite compaction and high temperature are the two most important factors that are expected to reduce microbial activity in the bentonite sealing layer of the DGR. However, the extent of microbial activity reduction with gradually increasing temperature and corresponding shifts in microbial community composition in a fully saturated bentonite system has not been adequately described. Another essential task is detecting the possible temperature threshold limiting microbial activity and proliferation in bentonite within the range of temperatures expected in the DGR. Similarly, the discrete effect of swelling pressure evolution upon saturation on microbial activity in bentonite remains unresolved. Therefore, this study aimed to (1) estimate the effect of swelling pressure as the single and critical parameter responsible for the reduction of microbial activity in compacted bentonite, (2) uncover shifts in microbial community composition due to pressure or temperature application demonstrating the extremophilic potential of indigenous microorganisms in bentonite, and (3) determine the possible temperature and pressure thresholds limiting microbial activity and proliferation in saturated bentonite.

## Materials and methods

### Bentonite

BCV is a Czech bentonite, mined and processed by Keramost Ltd. This form of bentonite is primarily composed of montmorillonite, with divalent exchangeable cations, mainly magnesium (Mg). The detailed characteristics of BCV have been provided elsewhere (Villar et al. 2020; Kašpar et al. 2021).

### Experimental set-up

Each experiment was performed on a suspension of BCV bentonite and sterile deionised water, with duplicate samples prepared for each treatment and sampling point. For each sample preparation, the bentonite was weighed directly into an experimental vessel (see below) and then transferred to an anaerobic glove box fed with an argon (Ar) atmosphere (O_2_ < 0.1 ppm) to deoxygenise for at least two weeks prior to the experiment. At the start of the experiment, sterile deionised water was added to each vessel to achieve a 1:5 (w/w) bentonite:water ratio. The vessel was then closed and manually shaken to homogenise the suspension.

For the pressure experiment, 100 mL volume polyetheretherketone (TecaPeek®; Ensinger, USA) cells were used as experimental vessels. Cells containing the homogenised suspension (see above) were connected to a pressure exchanger (Ar/anaerobic sterile deionised water) at low pressure (ca. atmospheric), after which the pressure was gradually increased to desired pressure levels, i.e. 10 MPa, 12 MPa or 15 MPa (maximum achievable pressure in the experimental system based on the cell design used), at laboratory temperature. Duplicate non-pressurised samples (atmospheric pressure applied only) were prepared and incubated in the same manner to serve as controls. After four weeks of pressure treatment, the cell lids were loosened to depressurise the samples, after which the suspensions were incubated at atmospheric pressure in a 94% Ar + 6% hydrogen (H_2_) atmosphere for another two- to three-weeks. This ‘regeneration period’ was included to assess possible recovery of microbial activity after pressure treatment. The pressure experiment sample was sequentially sampled after two weeks (herein denoted as 2w) and four weeks (4w) of pressure treatment, and again after the two- to three-week regeneration period (4 + 2/3w). The 2w samples are missing for the 12 MPa experiment due to COVID restrictions at the time of the experiment.

For the temperature experiment, the pre-prepared bentonite suspensions (see above) were placed into 200 mL DURAN glass vessels and exposed to four different temperatures (60, 70, 80 or 90 °C) in the integrated drying oven of an anaerobic box. Non-heated samples were also prepared and included as controls. After 4w of temperature treatment, the samples were incubated under laboratory temperature in a 100% Ar atmosphere for another 2/3w to detect possible recovery of microbial activity. The experiment at 90 °C was performed twice to confirm the results. In the second experiment, incubation during the 3w regeneration period was performed under a 94% Ar + 6% H_2_ atmosphere to boost microbial activity and better detect microbial recovery. The temperature experiment was sequentially sampled in the same manner as the pressure experiment (i.e. 2w, 4w and 4 + 2/3w).

### Sample processing

All sampling was performed in an anaerobic glove box. At each sampling point, the suspensions in the cells/vessels were manually shaken prior to opening for homogenisation. Next, 50 mL of each suspension sample was poured into a 50 mL sterile falcon tube and centrifuged for 10 min at 11,000x*g*. The resulting pellet (solid phase) was immediately stored in a freezer for subsequent genetic analysis (see "Genetic analysis" Section). Additionally, a 5 mL sample was also taken for cell extraction to enable microscopic detection of viable cells (see "Cell extraction and microscopic analysis" Section).

### Genetic analysis

#### DNA extraction and qubit measurement

DNA was isolated from each solid pellet (approximately 5 g wet weight) using the DNeasy PowerMax Soil Kit (Qiagen, Germany), following the manufacturer’s protocol. The resulting DNA extract (1 mL) was subsequently purified and concentrated to a final volume of 50 µL using the Genomic DNA Clean & Concentrator kit (Zymo Research, USA) and DNA concentration measured fluorometrically using a Qubit® 2.0 Fluorometer (Invitrogen, Life Technologies, USA), again following the manufacturer’s protocols. As microbial DNA is generally highly susceptible to environmental contamination during sample processing, a ‘kit (negative) control’ sample without the input matrix was processed in the same way as the real samples during each DNA isolation to uncover contamination arising during DNA isolation, either from the environment (laboratory background) or from the kits (kit contaminations).

#### Relative quantification by qPCR

Quantitative PCR (qPCR) on a LightCycler® 480 system (Roche, Switzerland) was used to monitor changes in the relative abundance of total bacterial biomass, using the universal primers U16SRT-F (5′-ACTCCTACGGGAGGCAGCAGT-3′) and U16SRT-R (5′ATTACCGCGGCTGCTGGC-3′) to target all bacteria encoding the V3 region of the 16S rRNA gene (Clifford et al. 2012). Preparation of the qPCR reaction mix and PCR cycler conditions were as described by (Shrestha et al. 2022), with two technical replicates analysed for each sample.

Obtaining fully reliable standards for complex environmental samples with a huge microbial (and resulting sequential) diversity is challenging, and the subsequent lack of a standardised calibration curve makes absolute quantification impossible. Hence, we applied relative quantification (RQ), where the relative abundance of the bacterial 16S rDNA gene was calculated and expressed as a fold change between two states (at a given sampling time and at an initial time in each sample) using the delta Cq method (Lhotský et al. 2021; Shrestha et al. 2021). This method uses amplification efficiency of the primer, determined by measuring the slope of curves constructed from a serial dilution of template DNA from five internal environmental standards. The Cq values are then normalised by the sample mass used for DNA extraction prior to calculation. Mean values of duplicate samples, together with their standard deviation, are used for data visualisation. A non-template control was also included in each qPCR run to check the background detection limit. Each sample was run in duplicate, with the resulting differences between duplicate Cq values always below 0.5.

#### Library preparation and 16S rRNA gene sequencing

For samples with a low DNA yield (< 0.5 ng/µL and Cq values > 18), two PCR reactions were performed with standard and barcoded fusion primers, while only one PCR reaction containing barcode fusion primers was performed in the case of samples with higher DNA concentrations (≥ 0.5 ng/µL and Cq values < 18). PCR conditions were as follows: an initial cycle at 95 °C for 3 min, followed by 10(first PCR)/35(second PCR) cycles at 98 °C for 20 s, 50 °C for 15 s and 72 °C for 45 s, with a final extension at 72 °C for 1 min. The thermocycling conditions were the same for both the first and second PCR reactions, except for a difference in the number of cycles. For both PCR runs, EliZyme HS HIFI MIX polymerase (Elizabeth Pharmacon, Czech Republic) and the universal primers 515 F (Dowd et al. 2008) and 802R (Claesson et al. 2010) were used for amplification of the hypervariable V4 region of the 16S rDNA gene. The size of the amplicon was kept below 400 bp to cover as much microbial diversity as possible *(*Němeček et al. 2017). The amplified PCR product was then purified using the Agencourt Ampure XP system at a 50:50 ratio of PCR product:Ampure XP paramagnetic beads (Beckman Coulter, USA), following the manufacturer’s protocol. The concentration of purified PCR product was measured using a Qubit 2.0 fluorimeter (Life Technologies, USA). Finally, barcode-tagged amplicons from different samples were mixed at equimolar concentrations (25nM solution in 20 µL) and sequencing performed on an Ion Torrent Personal Genome platform (Thermo Fisher Scientific, USA), using the Ion PGM Hi-Q Sequencing Kit with the Ion 314 Chip v.2 (Thermo Fisher Scientific, USA), following the manufacturer’s instructions.

#### Bioinformatics

The data obtained were processed using the QIIME 2 2021.8 software package (Bolyen et al. 2019). The raw sequence data were first demultiplexed and quality filtered using the q2-demux plugin, followed by denoising with DADA2 (*via* q2‐dada2) (Callahan et al. 2016). Taxonomy was assigned to Amplicon Sequence Variants (ASVs) using the q2‐feature‐classifier (Bokulich et al. 2018) and classified through classify-sklearn naïve Bayes against the Silva 138 database (Quast et al. 2013), after which mitochondria and chloroplasts were removed. Additionally, in the temperature experiment, the genus *Delftia* was removed as it was identified as a possible kit contaminant. This genus has previously been reported as a common contaminant in bentonite studies (Engel et al. 2019). Classification accuracy was evaluated against an artificial MOCK community sample and QIIME 2 outputs were processed using the phyloseq R package (McMurdie and Holmes 2013). Dissimilarity of bacterial communities between samples was visualised using Principal Coordinates Analysis (PCoA) with Bray-Curtis distance based on relative abundances (non-rarefied data). The same relative abundances were also used to plot taxonomy bubble plots, using only those bacteria with a mean relative abundance of > 0.005. Analysis of variance (ANOVA) and multivariate analysis of variance (MANOVA) were performed using the pairwise Adonis R package (Martinez Abizu 2020) to test for differences in microbial composition and sources of variability in samples treated under different experimental conditions. Kit controls and zero-point samples were omitted from these analyses to better distinguish between the effects studied.

### Cell extraction and microscopic analysis

Cell extraction was performed on selected samples using 5 mL of fresh suspension, as described by (Hlavackova et al. 2023). LIVE/DEAD (L/D) staining was applied to detect the presence of living and dead cells, using 8 µL of extract from each sample mixed with 4 µL of L/D BacLight ™ Bacterial Viability Kit fluorescent dye (Thermo Fischer Scientific, USA). The stained sample was incubated in the dark for 15 min before observing under a Zeiss Axio Imager M2 epifluorescence microscope (Carl Zeiss, Germany), using the AxioVision (AxioVs40 × 69 V v.4.9.1.0) imaging software program (Carl Zeiss, Germany).

As the red signal for dead cells/spores could potentially be masked by the autofluorescence of red stained bentonite particles, cell extraction was primarily used to confirm the presence of living, metabolically active cells in the samples, and to confirm microbial growth detected using the genetic methods described below. Unfortunately, direct cell counts were not possible due to the presence of dense particles formed during the extraction protocol (Hlavackova et al. 2023). Owing to incomplete homogenisation, a bentonite pellet was observed at the bottom of the experimental cells in the 10 MPa pressure experiment. Consequently, cell extraction was not performed from these samples, and it was only performed on completely homogenised samples from the 12 and 15 MPa experiments. In the temperature experiment, only the 60 and 90 °C samples were used for cell extraction due to time constraints.

## Results

### Pressure experiment

#### Cell extraction

**Fig. 1:**
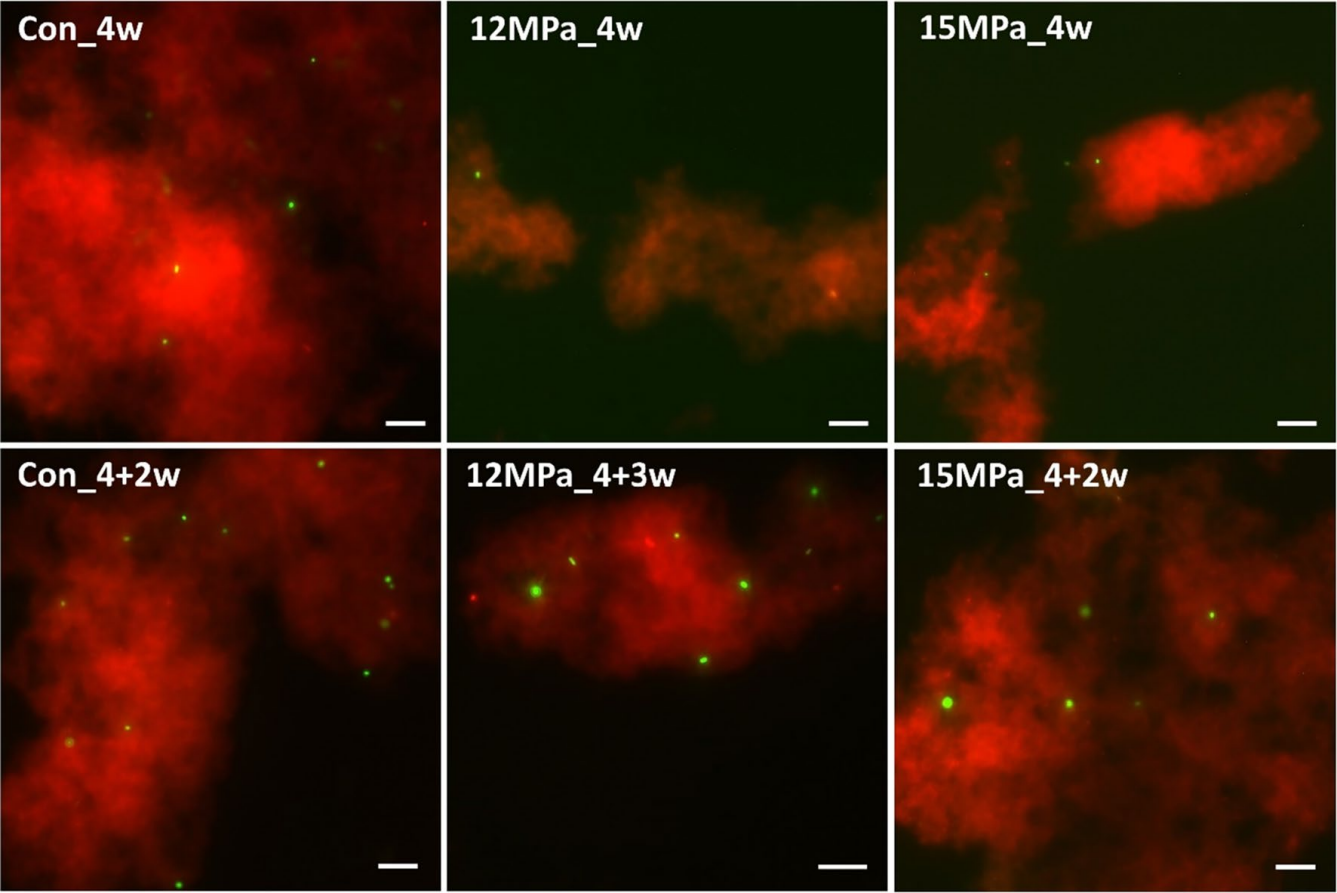
L/D stained cell extracts from bentonite suspensions exposed to atmospheric pressure (Con), 12 and 15 MPa for 4 weeks (4w) and after a regeneration period of 3 weeks (3w; 12 MPa), or 2 weeks (2w; 15 MPa). Green = live cells, red = dead cells and bentonite

The L/D stained extracts from the bentonite suspensions after 4w treatment and further 2/3w regeneration period are visualized in the Fig. 1. The presence of live cells was detected in all samples pressurised at 12 and 15 MPa and their relevant controls.

#### Genetic analysis

**Fig. 2:**
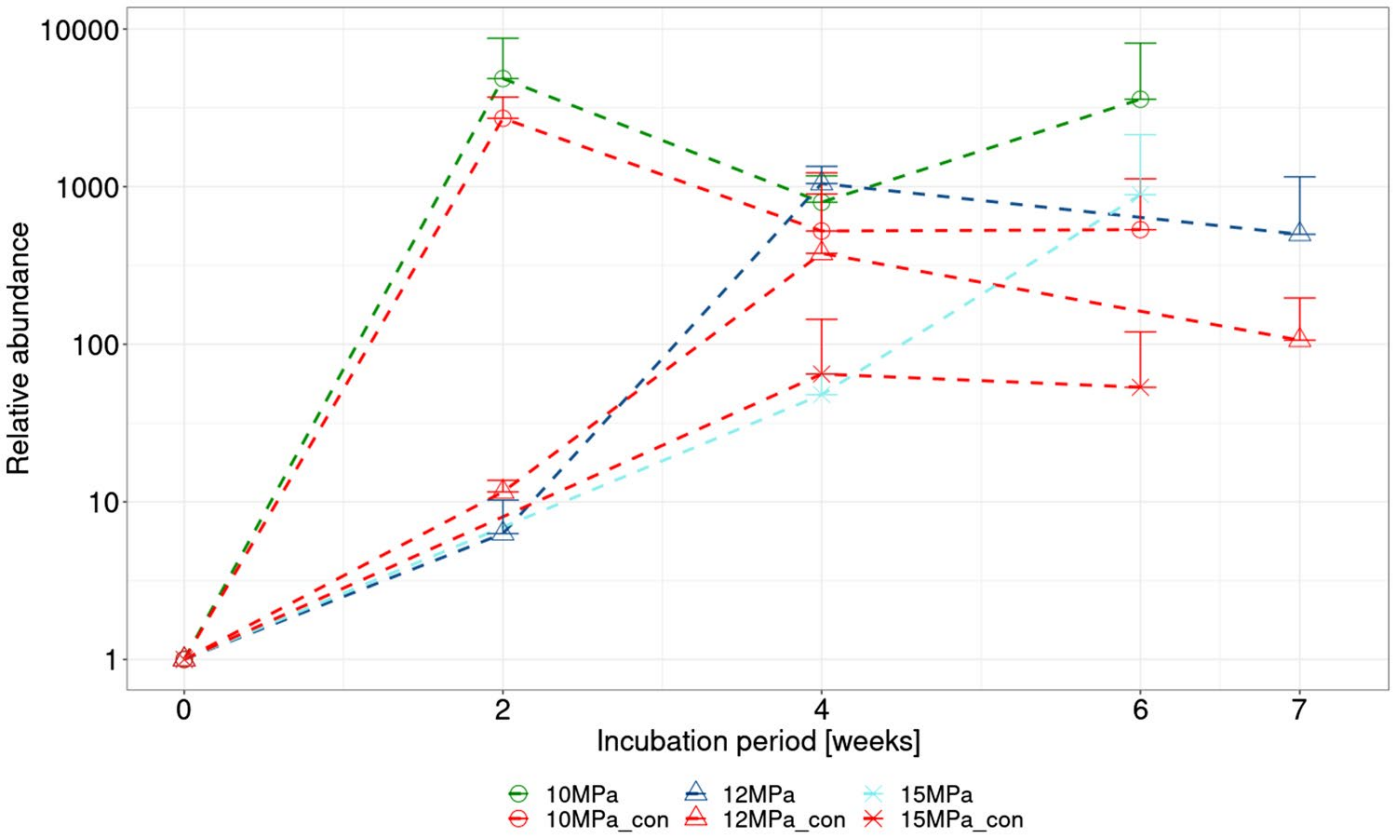
Relative quantification of microbial abundance detected in samples pressurised at 10, 12, and 15 MPa and their respective controls exposed to atmospheric pressure (con), based on total microbial biomass detected using the 16S rRNA gene. Mean values of duplicate samples, together with their standard deviation, are shown for each sample type and sampling time

After 4w exposure, qPCR analysis indicated an increase in total microbial biomass in all samples, irrespective of the pressure applied (based on relative abundance of the 16S rRNA gene). Subsequently, relative microbial abundance stagnated, or even declined, in all samples over the following 2/3w regeneration period, except for the sample pressurised at 15 MPa (Fig. 2).

**Fig. 3:**
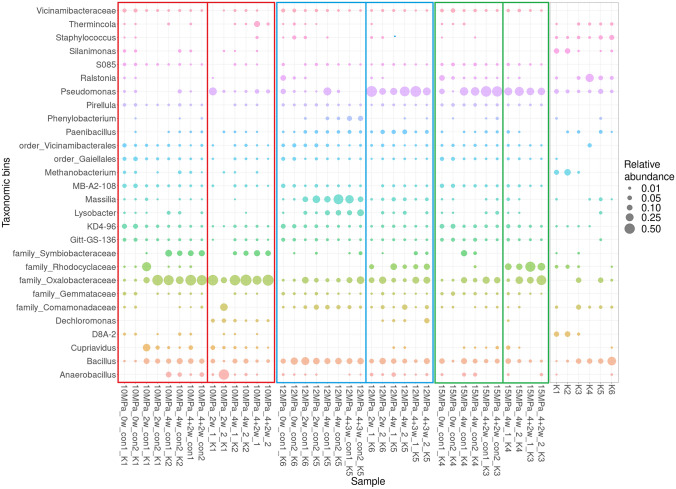
Microbial community profiles (ASV grouped at genus level) of samples pressurised at 10, 12 and 15 MPa and their respective controls exposed to atmospheric pressure (con). 0w/2w/4w/4 + 2w/4 + 3w = weeks of incubation. A co-isolated kit control (K1-K6) is listed at the end of each sample name. Only those genera at or above 0.5% relative abundance are shown; missing bubbles denote relative abundances < 0.1%

Sequencing results showed the presence of a diverse microbial community in all samples, though samples from the 10 MPa pressure experiment had a slightly different pattern than those from the 12 and 15 MPa experiments (Fig. 3). Samples from the 10 MPa experiment were dominated by unspecified representatives of the families *Oxalobacteraceae* and *Symbiobacteraceae*, along with the common genera *Anaerobacillus, Bacillus, Cuprivavidus* and *Pseudomonas* (Fig. 3). No significant differences (ANOVA, p > 0.05) were detected between pressurised and control samples in the 10 MPa experiment. On the other hand, significant differences in microbial composition were detected between pressurised and control samples from the 12 MPa (p = 0.005) and 15 MPa experiments (p = 0.027; Fig. 3). Control samples from the 12 MPa experiment were dominated by the genera *Bacillus, Lysobacter* and *Massilia*, along with representatives of the family *Oxalobacteraceae*, while the 15 MPa control samples were dominated by the genera *Bacillus* and *Pseudomonas*, together with members of the *Oxalobacteraceae *family. In comparison, samples pressurised at 12 and 15 MPa were dominated by the genus *Pseudomonas* and representatives of the *Rhodocyclaceae *and *Oxalobacteraceae *families, along with the genus *Bacillus (*Fig. 3).

Microbial composition was generally consistent in the duplicate samples, confirming the reliability of the genetic signal. Kit control signals were somewhat stochastic, though some genera, such as *Bacillus, Pseudomonas* and *Staphylococcus*, were detected in all controls (Fig. 3), and as such possibly represent the laboratory background signal or kit contaminants. The kit control signals may also have been influenced by sequencing bias as, in all cases, they represented very-low DNA samples with yields below the detection signal.

**Fig. 4:**
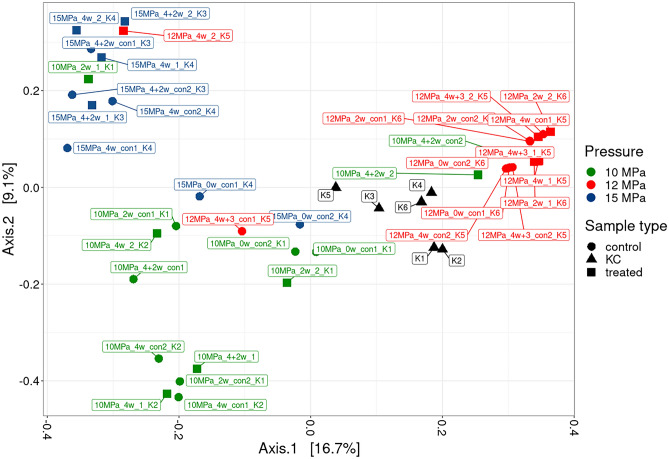
Principal-coordinate analysis (PCoA) ordination plot based on Bray-Curtis distance metrics, showing grouping of the pressure experiment samples (samples pressurised at 10, 12 and 15 MPa and their respective controls exposed to atmospheric pressure - con), KC = kit control samples (denoted as K1-K6). The percentage of variability explained by each axis is denoted in square parentheses

PCoA analysis revealed three separate clusters roughly indicative of each pressure experiment, with the kit control samples clustered in between (Fig. 4). This clustering was further verified by MANOVA analysis, which confirmed a statistically significant effect of experimental batch (p = 0.001) explaining 34% of variability in the data. Differences between the control and treatment samples were also statistically significant (p = 0.001) and explained a further 9% of variability, which corresponds well with the ANOVA results described above.

### Temperature experiment

#### Cell extraction

**Fig. 5:**
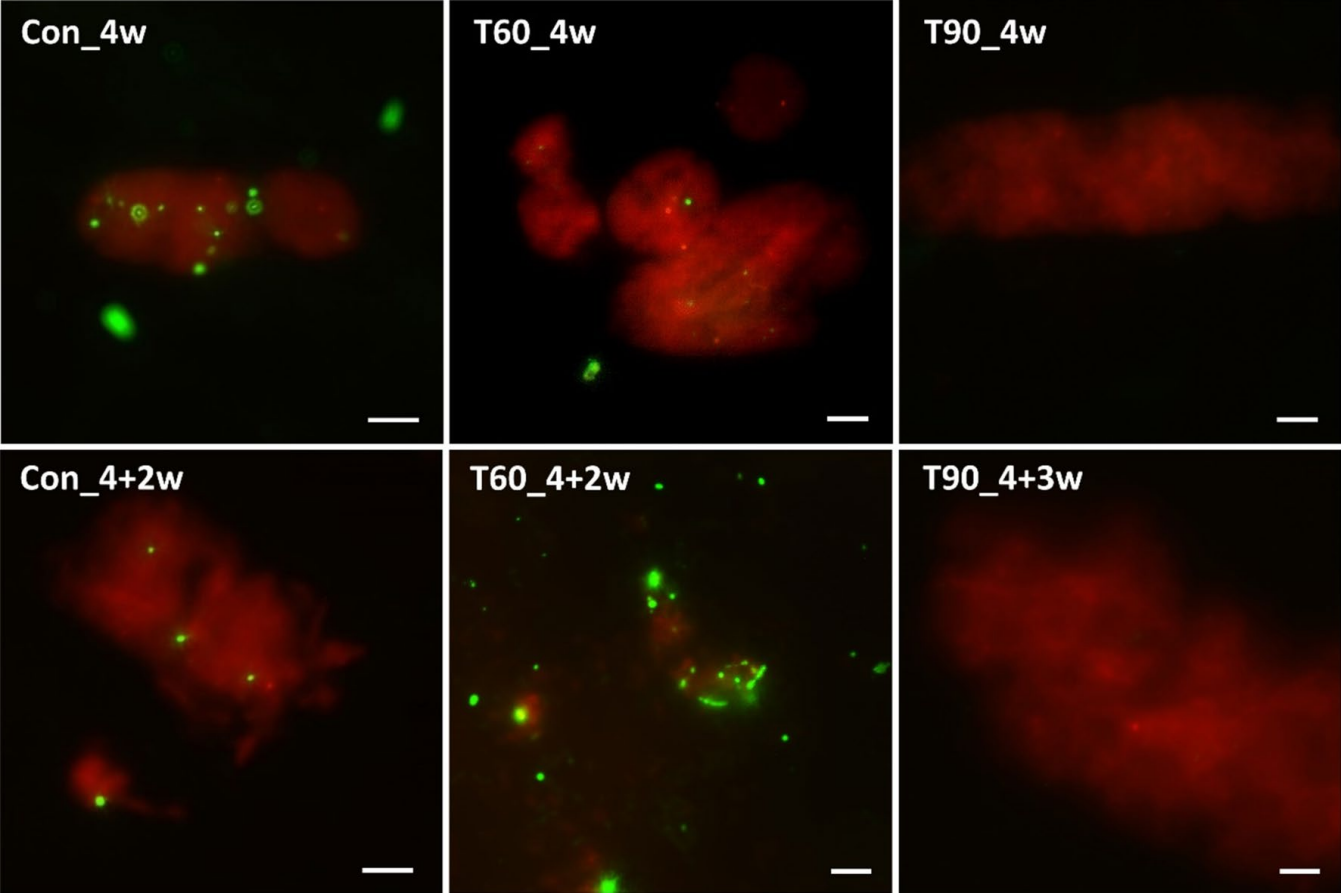
L/D stained cell extracts from control and samples heated to 60 °C (T60) and 90 °C (T90) after four weeks (4w) exposure and a subsequent regeneration period of two weeks (2w) or three weeks (3w) at laboratory temperature. Con = control samples kept at laboratory temperature, T = temperature applied, green = live cells, red = dead cells and bentonite particles. Scale bar sizes 10 μm

The L/D stained extracts showed a substantial decrease in viable cells with increasing temperature applied after 4w heat treatment and a further 2/3w regeneration period (Fig. 5). While living cells were observed after both 4w exposure and the 4 + 2w regeneration period in samples heated to 60 °C (and the corresponding controls), very few cells (these may even have been artefacts, such as minerals binding the dye) were observed after 4w in samples heated to 90 °C, and no living cells at all after the 4 + 2w regeneration period (data not shown). Similar results were also observed in the second 90 °C experiment with a 3w regeneration period, which was performed in an anaerobic glove box with H_2_ as an energy substrate to boost microbial recovery (Fig. 5), thereby confirming a lack of microbial proliferation in samples heated to 90 °C.

#### Genetic analysis

**Fig. 6:**
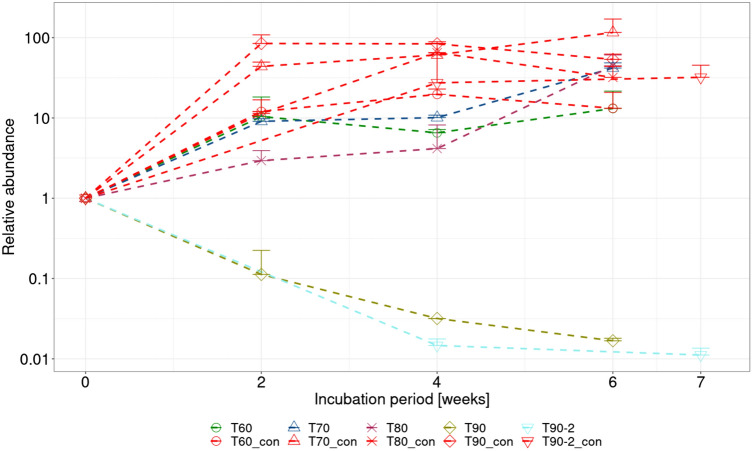
Relative quantification of microorganisms by qPCR in the temperature experiment (samples heated to 60 °C (T60), 70 °C (T70), 80 °C (T80) and 90 °C (T90) and their respective controls kept at laboratory temperature (con)), based on total microbial biomass detected using the 16S rRNA gene. Mean values of duplicate samples, together with their standard deviations, are shown for each sample type and sampling time

qPCR unambiguously demonstrated a gradual decrease in total microbial biomass with increasing incubation temperature (Fig. 6). Furthermore, lower relative microbial abundances were observed in all heated samples compared to their respective controls (Fig. 6). Samples heated to 60 or 70 °C showed a small increase in relative abundance with time during the 4w heating period, followed by a more pronounced increase during the 2w regeneration period, with growth during the regeneration period more pronounced in samples heated to 70 °C. In samples heated to 80 °C, there was a negligible change in relative microbial abundance over the 4w heating period, but a marked increase in biomass during the regeneration period. In contrast, there was a gradual decrease in relative abundance over the 4w heating period in samples heated to 90 °C, which then continued throughout the regeneration period (Fig. 6). A similar pattern was also observed during the second 90 °C confirmation experiment, which was performed under more favourable conditions (Ar + H_2_ atmosphere) over a prolonged incubation period. This suggests that 80 °C can be considered as the limiting temperature for microbial activity, and 90 °C the limiting temperature for both microbial activity and regeneration after heat treatment. In comparison, all control samples showed the same general trend of a rapid increase in relative microbial abundance over the first two weeks, with levels remaining relatively stable over the rest of the experiment, aside from natural variability between samples (Fig. 6).

**Fig. 7:**
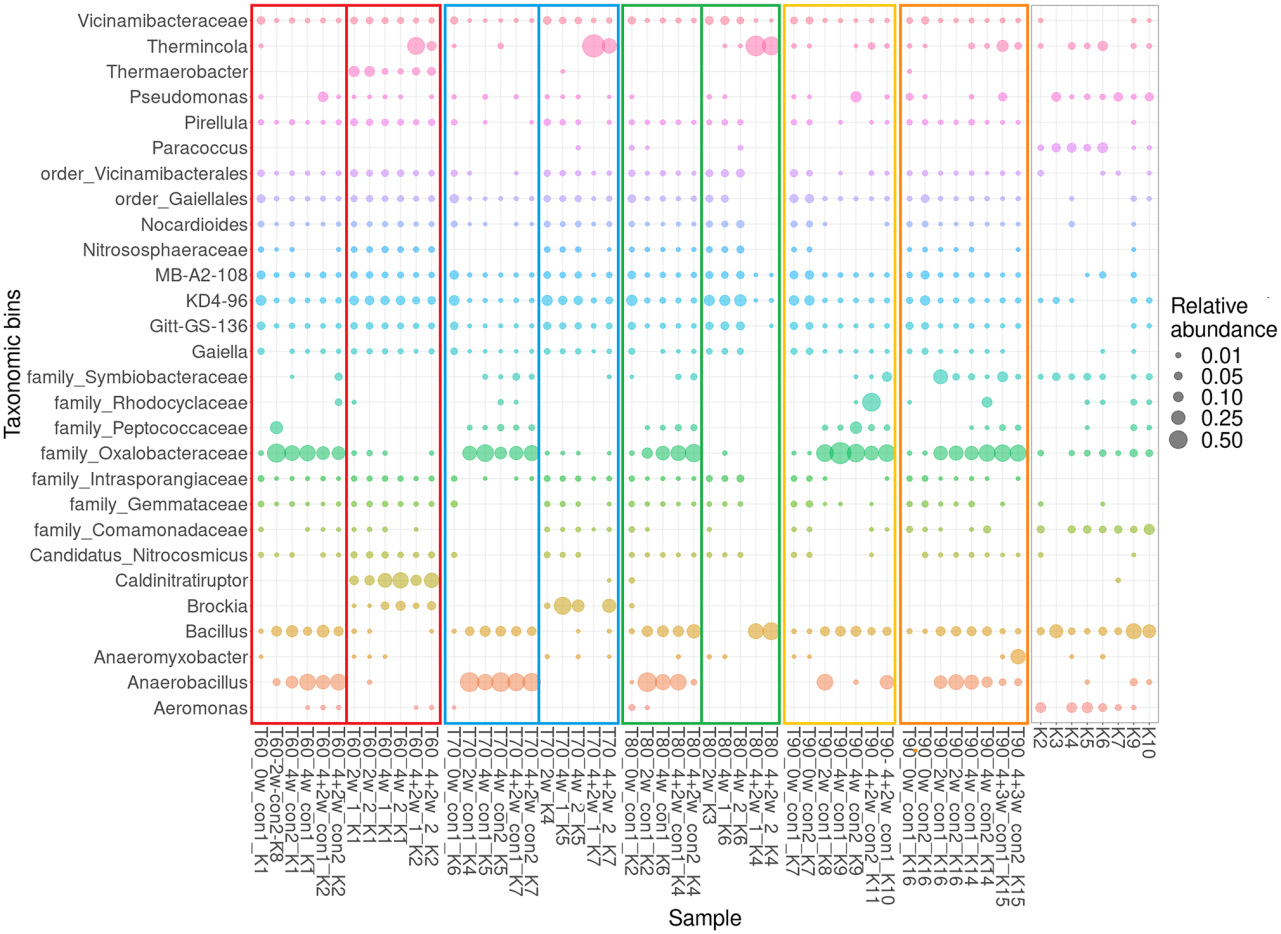
Microbial community profiles (ASV grouped at genus level) of samples heated to 60 °C (T60), 70 °C (T70), 80 °C (T80) and 90 °C (T90) and their respective controls kept at laboratory temperature (con). 0w/2w/4w/4 + 2w/4 + 3w = weeks of incubation. A co-isolated kit control (K1-K10) is listed at the end of each sample name (K1 & K8 were excluded due to the low number of sequences obtained). Only those genera at or above 0.5% relative abundance are shown, missing bubbles denoting relative abundance < 0.1%

Sequencing analysis indicated both diverse community structures and a strong effect of heat treatment on microbial community composition. Microbial composition was very similar in all non-heated control samples, regardless of the experimental batch, with the nitrate-reducing bacterial genera *Anaerobacillus, Bacillus* and *Pseudomonas* dominating alongside representatives of the Oxalobacteraceae family (Fig. 7). In the heated samples, however, microbial community composition differed according to the temperature applied. Samples heated to 60 °C showed proliferation of the thermophilic genera *Caldinitratoruptor, Brockia* and *Thermaerobacter* and a significant increase in relative abundance of the iron-reducing bacterial genus *Thermincola* over the 2w regeneration period. In samples heated to 70 °C, the genus *Brockia* dominated, with *Thermincola* becoming more prominent during the regeneration period, as in the 60 °C sample. No obvious proliferation was observed in samples heated to 80 °C, though the genera *Thermincola* and *Bacillus* came to dominate during the regeneration period. Samples heated to 90 °C could not be sequenced as almost no amplifiable DNA were obtained after DNA extraction, which corresponds well with the qPCR analysis. Likewise, no proliferation was observed during the regeneration period, suggesting that 90 °C represents the limiting temperature for microbial growth and proliferation in BCV suspensions.

The duplicate samples showed consistent microbial compositions, confirming reliability of the genetic signal. As in the pressure experiment, kit controls contained genera such as *Bacillus*, *Pseudomonas, Aeromonas* and *Paracoccus*, or representatives of the families Oxylobacteraceae, Symbiobacteraceae and Commamonadaceae (Fig. 7), which most probably represent laboratory background signals or kit contamination. Sequencing bias is also a possibility as all DNA yields for the controls were below the detection limit. The total sequence read counts for each sample are included in Supplementary Table 1.

**Fig. 8:**
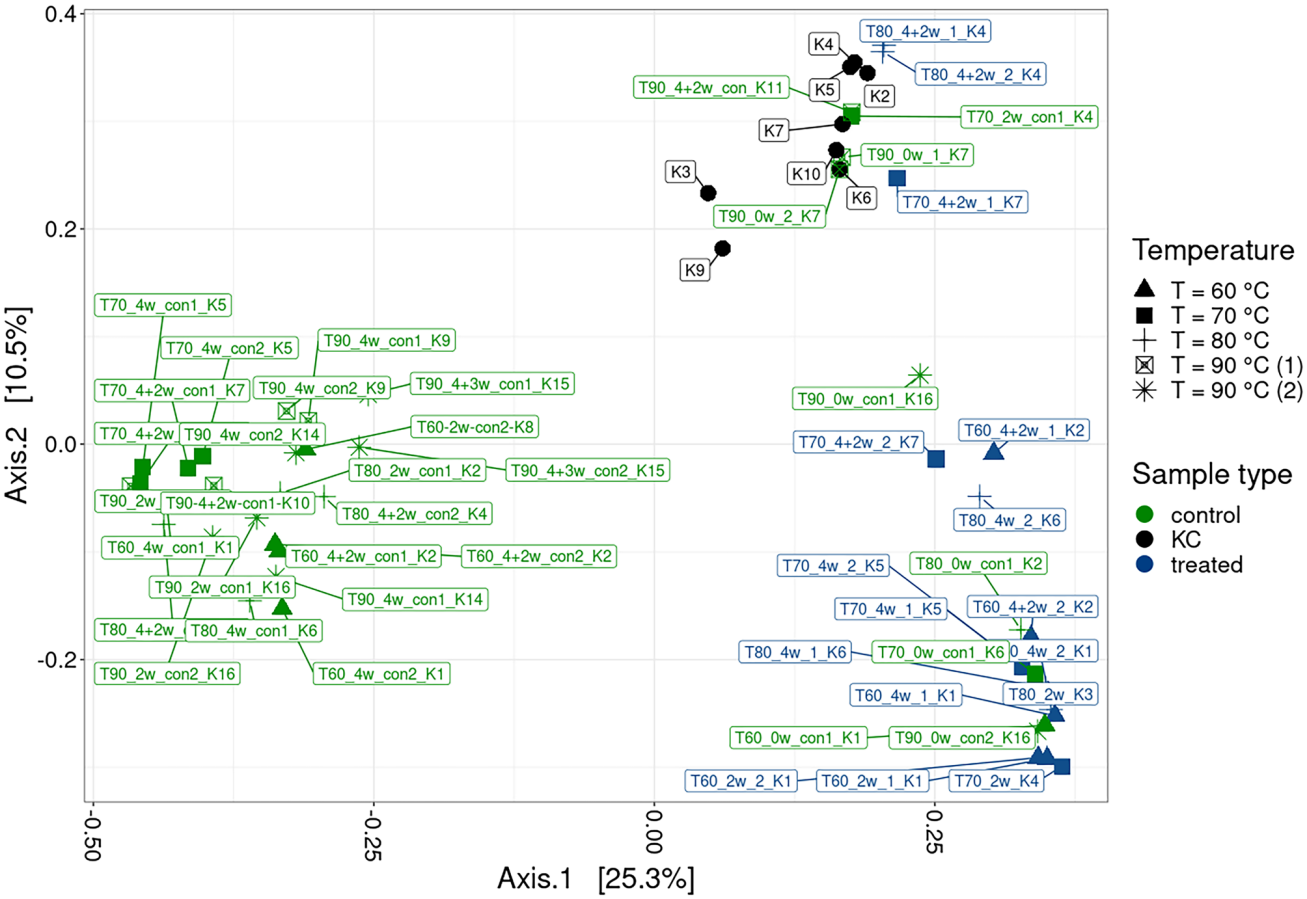
Principal-coordinate analysis (PCoA) ordination plot based on Bray-Curtis distance metrics, showing the grouping of samples from the temperature experiment (samples heated to 60 °C (T60),70 °C (T70), 80 °C (T80) and 90 °C (T90) and their respective controls kept at laboratory temperature (con), KC = kit control samples (denoted as K2-K17). The percentage of variability explained by each axis is denoted in square parentheses

PCoA indicated that most control samples were clustered together, with kit control samples forming a second cluster together with some of the samples heated to 70 or 80 °C, indicating that these samples exhibited a relatively weak signal comparable to the background detected by the kit controls (Fig. 8). A third cluster was formed mainly of samples heated to 60 or 70 °C, with ongoing microbial activity confirmed by qPCR and microscopic analysis.

## Discussion

### Effect of pressure

Our study showed that a four-week application of pressure up to levels corresponding to the swelling pressure of saturated compacted bentonite of high dry density does not considerably inhibit microbial growth or viability to any great degree in bentonite suspensions. Microbial growth in pressurised samples was similar to that in control samples, regardless of the pressure applied (up to 15 MPa). Potts (1994), in summing up previous data on turgor pressure in prokaryotic cells, reported pressures of between 0.08 MPa and 2 MPa in most cells examined. For this reason, 2 MPa or 5 MPa is repeatedly mentioned as a possible swelling pressure limiting value for microbial growth and proliferation in compacted bentonite (Pedersen 2017). However, our results have shown that this assumption is probably not correct, as a 4w application of 15 MPa pressure did not reduce microbial activity or proliferation in bentonite suspensions. Though the experimental duration was short compared to the expected DGR life time (up to 1 My, King and Kolář 2018), very short generation time of bacteria (< 1 h) in many common species (Madigan et al. 2018) together with their high sensitivity to environmental conditions resulting rapid shifts in microbial composition observed in many laboratory and field studies (e.g. Brown et al. 2015; Miettinen et al. 2015), Based on the changes observed, we conclude that factors other than swelling pressure, such as water activity and/or availability, nutrient diffusion limitation or restricted pore size (Pedersen 2017), must be considered as key drivers of microbial activity inhibition in saturated compacted bentonite.

Interestingly, we detected a strong effect of experimental batch on microbial composition, though relative microbial abundance remained comparable between batches. A possible explanation may be the unintended formation of compacted bentonite plugs at the bottom of the experimental cells in all samples from the 10 MPa experiment. As it was not technically possible to run all three pressure experiments simultaneously during this study, each was run separately, with the 10 MPa experiment performed first. The bentonite plug was probably formed due to incomplete homogenisation during preparation of the suspension. Subsequently, plug formation could have lowered microbial activity response to pressure application, resulting in the similarity between microbial composition in the 10 MPa pressurised and control samples. In comparison, the samples from the 12 and 15 MPa experiments were fully homogenised as the process had been optimised prior to sample preparation. Subsequently, we detected significant differences in microbial community composition between the controls and pressurised (12 and 15 MPa) samples, including promotion of the family Rhodocyclaceae and genus *Pseudomonas* or *Dechloromonas* (Fig. 3), indicating that application of pressure can indeed influence microbial community composition, while not limiting overall microbial abundance.

### Effect of temperature

Heat treatment strongly influenced microbial activity and survivability in bentonite suspensions, with the proliferation of several thermophilic bacteria, such as *Caldinitratiruptor* or *Brockia*, detected at temperatures up to 70 °C. Furthermore, other thermotolerant spore-forming genera, such as *Thermincola* or *Bacillus*, exhibited high-temperature tolerance and an ability to rapidly proliferate from dormant stages over the regeneration period. This clearly demonstrated the potential of indigenous bentonite bacteria to remain metabolically active at elevated temperatures, and the ability of spore-forming bacteria to regain metabolic activity when conditions become more favourable (Nicholson et al. 2000; Stroes-Gascoyne 2010). Previous studies have demonstrated that similar opportunistic microorganisms, including sulphate reducers, iron reducers or nitrate reducers, are also capable of surviving heat treatment of bentonite (Aoki et al. 2010; Gilmour et al. 2022).

The temperature experiment further showed that 90 °C represents the limiting temperature for microbial activity and proliferation in bentonite suspensions. While the control samples clearly demonstrated microbial proliferation increasing with incubation time, microbial presence was not detected in bentonite suspensions directly after heating to 90 °C or at the end of the regeneration period, the same result being obtained by both microscopic and genetic methods. This suggests that microbial survivability, including that of thermotolerant spore formers, is hindered, or even completely inhibited, in bentonite suspensions heated to 90 °C. This result corresponds well with previous temperature experiments performed on compacted bentonite, where a considerable decrease in total microbial abundance was observed at higher temperatures (> 70 °C) under realistic repository conditions (Pedersen et al. 2000; Lydmark and Pedersen 2011).

Exposure to high temperatures can destroy microbial cellular proteins, membrane lipids and nucleic acids, making them unstable and limiting their survivability, with subsequent undesirable physical or chemical changes to the bacterial cells (Wolf et al. 1989; Trevors 1996). Heat resistance will depend on critical factors such as sample type, heat exposure duration, the actual temperature (Otte et al. 2018) and, importantly, moisture content.

While soil microorganisms are generally able to survive heat relatively well in dry soil, where the bacteria remain in the spore form, microbial heat resistance is generally reduced upon wetting and germination (Dunn et al. 1985; Nicholson et al. 2000). In a repository environment, bentonite temperatures may become temporarily high, though they are unlikely to exceed 100 °C (Johnson et al. 2002; Bennett and Gens 2008). Consequently, the saturation level will gradually evolve depending on the original water content of the compacted bentonite upon deposition, temperature evolution, and the bentonite’s dry density (Hökmark 2004). Our experiment demonstrated that survivability of microorganisms is severally hindered at elevated temperatures in a fully saturated environment; thus, evolution of bentonite water saturation close to the canister during the initial hot stage in the repository may prove to be a crucial parameter. If the bentonite layer remains largely unsaturated, and the microorganisms remain in a dormant state, microbial heat resistance may remain relatively high (Nicholson et al. 2000; Setlow 2014). Further experiments with varying temperatures and moisture contents, undertaken at different time scales and under realistic repository conditions, will be needed to reliably predict the evolution of microbial activity in bentonite sealing layers.

## Conclusions

Our study showed that a 4w application of hydrostatic pressures up to 15 MPa, corresponding to a compacted bentonite swelling pressure of ca. 1700 kg/m3, did not to inhibit microbial activity in BCV bentonite suspensions. Thus, our short-term experiment implies that swelling pressure solely should not be considered the key factor responsible for limiting microbial activity in compacted bentonite. Further long-term studies will be needed to confirm this, and examine other factors, such as water activity and availability, nutrient diffusion limitation or restricted pore size, that could be responsible for variations in compacted density necessary to limit microbial activity in different bentonites.

Conversely, temperature showed a strong effect on microbial activity and survivability in bentonite suspensions. While several active thermophilic microbial species were detected in samples heated up to 70 °C, confirming the potential of microbes to survive elevated temperatures in bentonite, 90 °C was identified as limiting to microbial survivability. As bentonite suspensions represent a fully saturated environment, and microbial heat resistance is influenced by water content, further experiments examining different temperatures and moisture contents under realistic repository conditions will be needed to reliably predict the evolution of microbial activity in bentonite at different stages of DGR evolution. Furthermore, our laboratory experiments using bentonite suspensions will need further verification under more realistic long-term laboratory or *in-situ* experiments using compacted bentonite. The findings of our study have significant implications for environmental safety as it helps to understand better the extent of microbial activity that could impact repository integrity and identify strategies that could mitigate the microbial potential within DGR. From an environmental safety perspective, the study emphasizes the need to consider microbial activity when assessing long-term repository stability.

### Supplementary Information

Below is the link to the electronic supplementary material.

Supplementary material 1 (XLSX 25.9 kb)

## Data Availability

Sequencing data are openly available in the NCBI database (BioProject ID: PRJNA967163); qPCR raw data are openly available in the Zenodo repository (10.5281/zenodo.8079580), together with microscopic source images from temperature and pressure experiments (10.5281/zenodo.8079808 / 10.5281/zenodo.8077992).
